# Automated Billing Code Retrieval from MRI Scanner Log Data

**DOI:** 10.1007/s10278-019-00241-z

**Published:** 2019-06-25

**Authors:** Jonas Denck, Wilfried Landschütz, Knud Nairz, Johannes T. Heverhagen, Andreas Maier, Eva Rothgang

**Affiliations:** 1 Institute of Medical Engineering, Technical University of Applied Sciences Amberg-Weiden, Weiden, Germany; 2 Pattern Recognition Lab, Department of Computer Science, Friedrich-Alexander University Erlangen-Nürnberg, Erlangen, Germany; 3 Siemens Healthineers, Erlangen, Germany; 4 Department of Diagnostic, Interventional, and Pediatric Radiology, Inselspital, University of Bern, Bern, Switzerland

**Keywords:** Machine learning, Magnetic resonance imaging, Medical coding, Reimbursement, Workflow enhancement

## Abstract

Although the level of digitalization and automation steadily increases in radiology, billing coding for magnetic resonance imaging (MRI) exams in the radiology department is still based on manual input from the technologist. After the exam completion, the technologist enters the corresponding exam codes that are associated with billing codes in the radiology information system. Moreover, additional billing codes are added or removed, depending on the performed procedure. This workflow is time-consuming and we showed that billing codes reported by the technologists contain errors. The coding workflow can benefit from an automated system, and thus a prediction model for automated assignment of billing codes for MRI exams based on MRI log data is developed in this work. To the best of our knowledge, it is the first attempt to focus on the prediction of billing codes from modality log data. MRI log data provide a variety of information, including the set of executed MR sequences, MR scanner table movements, and given a contrast medium. MR sequence names are standardized using a heuristic approach and incorporated into the features for the prediction. The prediction model is trained on 9754 MRI exams and tested on 1 month of log data (423 MRI exams) from two MRI scanners of the radiology site for the Swiss medical tariffication system Tarmed. The developed model, an ensemble of classifier chains with multilayer perceptron as a base classifier, predicts medical billing codes for MRI exams with a micro-averaged F1-score of 97.8% (recall 98.1%, precision 97.5%). Manual coding reaches a micro-averaged F1-score of 98.1% (recall 97.4%, precision 98.8%). Thus, the performance of automated coding is close to human performance. Integrated into the clinical environment, this work has the potential to free the technologist from a non-value adding an administrative task, therefore enhance the MRI workflow, and prevent coding errors.

## Introduction

Medical coding can best be described as the translation of unstructured, medical information into a series of codes, with each code representing a certain diagnosis or procedure [1]. It can be a tedious and time-consuming task, which requires special training in medical coding for the used medical code set. Medical diagnoses are primarily encoded with the International Statistical Classification of Diseases and Related Health Problems (ICD). In addition to medical diagnosis coding, medical services and procedures are encoded in procedure billing codes that are used for a standardized billing process and procedure documentation. Thus, procedure coding is the basis for the reimbursement for any medical examination. Different countries use their own procedure billing code set; for instance, the Current Procedural Terminology (CPT) used in the USA, the “einheitliche Bewertungsmaßstab” and the “Gebührenordnung für Ärzte” in Germany or Tarmed in Switzerland [2]. Tarmed, from French **tar**if **méd**ical, is the fee-for-service tariff system for all outpatient services in Switzerland, comprising more than 4600 different billing codes. Each code describes a medical procedure or service that is associated with a technical and a professional component, which determine the reimbursement for the code. Tarmed codes are therefore used to standardize and exchange billing information between health care provider and insurance [2]. All outpatient medical services and procedures are encoded in these tariffs, but Tarmed is also used for certain services within inpatient care (e.g. radiology services).

Although Tarmed comprises an extensive code set, not all procedures that are routinely performed are necessarily encoded in distinct billing codes. New examination techniques evolve fast, but the process to encode a new technique or procedure into the tariff system is slow [3]. In order to get reimbursed for services that are not yet encoded (but are already included in the service catalogue for Swiss compulsory health insurance), codes with similar associated complexity in terms of workload and costs to the conducted procedure can be charged. This is called analogous coding, which does not necessarily follow fixed rules and can differ through the coding practice of each site.

The goal of this paper is to develop an algorithm that can reliably predict Tarmed medical billing codes (for Tarmed version 01.08.01). Most work on clinical coding automation has not reached acceptable performance yet (see the “Related work” section). In our work, we narrow our focus on MRI exams and use internal log data from MRI scanners (MRI log data) as data basis. MRI log data provide information about the set of executed MR sequences, MR scanner table movements, and given a contrast medium. The information available in MRI log data is comparable to non-patient-related DICOM metadata for technical information. However, MRI logs offer an enhanced set of attributes that are unavailable in the radiology information system (RIS) or Picture Archiving and Communication System (PACS) [4], such as an extensive set of MRI acquisition parameters and MRI scanner table movements that can be used for feature extraction for the prediction task.

A large amount of data and the fact that information about billing codes cannot be directly retrieved from the MRI log data, but rather exists in form of patterns, facilitate the use of a machine learning model. Additionally, not all MRI procedures are encoded in clearly defined billing codes (analogous coding), which also supports the use of a machine learning approach.

We evaluated our approach on data from two MRI scanners (Siemens MAGNETOM Aera and Siemens MAGNETOM Skyra) of a single hospital university site. In the current workflow of this site, a technologist enters the conducted services (in form of site-specific exam codes) and the Tarmed billing codes for an MRI exam in the RIS, usually during or at the end of the exam. A set of Tarmed codes is associated with each entered exam code (e.g. the exam codes corresponding to “MR Hip” and “MR-guided biopsy”). An exam code is preregistered with the respective Tarmed codes in the RIS, but additional billing codes can be manually added or removed, depending on the performed MRI exam. Moreover, the billing codes for each radiological exam are reviewed by the responsible person for procedure coding at the hospital.

Collected coding data represent codes reported by the technologists, but not the final billing codes submitted to the insurer. One goal of this work was to identify potential flaws of the procedure coding workflow for MRI exams. By using the coding data as reported by the technologists, we were able to assess the error rate of technologists. The final billing codes submitted to the insurer were not available as this process can take several months and final billing codes are compiled and submitted outside the RIS of the hospital site. However, in order to evaluate the developed automated coding algorithm properly, we generated a ground truth billing code set used for testing.

The rest of the paper is organized as follows: the “Related work” section presents related work on automated medical coding and the utilization of log data from imaging modalities. In the “Methods” section, we present the used dataset and describe the prediction model, including feature extraction, feature processing, and classification methods. Moreover, we describe ground truth data generation and the reported evaluation metrics. In the “Results” section, we present our assessment of the performance of automated coding and manual billing code assignment (manual coding) through the technologists. The “Discussion” section discusses the presented results, and the paper concludes with the “Conclusion” section.

## Related work

Automated medical code assignment has been an active field of research in recent years. Perotte et al. [5] studied the use of hierarchy-based support vector machines (SVMs) for the assignment of ICD-9 (ninth revision) codes to discharge summaries. In their results, they reported a F1-score of 39.5%. They furthermore discovered that the supposedly ground truth codes are not perfect, and therefore their measures, such as recall and precision, are likely underestimated. However, they have not assessed the error rate within their dataset and consequently, their true performance cannot be determined precisely. Kavuluru et al. [6] evaluated supervised learning approaches for the assignment of ICD-9-CM (clinical modification) to electronic medical records (EMR) dataset. They experimented with different problem transformation approaches (including binary relevance and ensemble of classifier chains) and reported a micro F1-score of 48% overall codes with at least 50 training examples for the dataset of EMRs containing 1231 labels in total. Atutxa et al. [7] used diagnostic terms to retrieve information from Electronic Health Records (EHR). The correct ICD code among more than 1500 possible ICD codes was found with 92% precision for the main disease.

Moreover, earlier attempts on automated medical coding include a hierarchical approach for ICD-9 code assignment to medical documents [8], the use of ensemble learning for the assignment of ICD-9-CM codes to the clinical history and impression sections of radiology reports [9], information retrieval from search engines, boosting as well as rule-based approaches [10].

Procedure codes from ICD-10 were predicted from the clinical narratives using several levels of abstraction in [11] with a F1-score of 48.5%.

Event logs from imaging modalities were used in [12] to retrieve the examined body region in MRI exams from sequence parameters with a classification accuracy of 94.7% and in [13] to classify interventional X-ray exams into respective procedures or examined anatomy, reaching a classification accuracy of 92.7%.

To summarize, it can be stated that a large part of the research has focused on the prediction of diagnosis codes [5–10] rather than on procedure billing codes [11]. Additionally, the data basis of the published methods for predicting medical codes usually consisted of discharge summaries and free-text reports, although there were recent attempts to utilize MRI log data for data analytic purposes [12, 13]. Thus, this paper is one of few works on automation of procedure coding, and to the best of our knowledge, it is the first attempt to focus on the prediction of procedure billing codes from data excluding EMRs or EHRs. Billing code prediction is a sensitive task since errors due to overcoding, i.e. reporting medical services wrongly, or undercoding, i.e. not reporting rendered services, lead to associated inaccuracies in revenue streams that must be prevented.

## Methods

### Data

The MRI log data contain information about the set of conducted sequences and associated MR sequence parameters, approximate age of the subject, information about the MRI scanner table movements, and contrast medium.

The billing codes are extracted from the RIS with an SQL query. The codes were entered by the technologists and comprise Tarmed codes for each MRI exam that serve as target data for training the algorithm.

The datasets were stripped of any patient-identifiable information, compliant with Swiss personal data protection laws.

### Data merging and cleaning

**Fig. 1:**
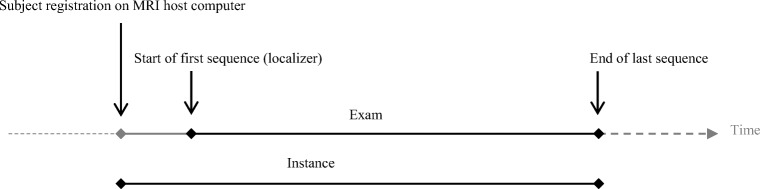
Time line of an MRI exam. All logs from the subject registration on the MRI host computer to the end of the last sequence describe a single instance

First, the MRI logs were merged with the billing code data via the associated time stamps and assigned to a single instance, i.e. an MRI exam for a single subject. Logs associated with a subject comprise the data from the time of the subject registration on the MRI host computer to the end of the last MR sequence (see Fig. 1).

The codes also comprise non-imaging-related billing codes. These non-imaging-related codes were excluded, as they were not predictable with the available MRI log data. These codes include a code for establishing intravenous access through the technologist, a surcharge code for narcotized patients, or a physician’s service in absence of the patient. Furthermore, technical base service codes are added in the hospital information system, depending on the patient being out- or (narcotized) inpatient. At the investigated radiology site, no information about the status of the patient’s hospital stay (out-/inpatient) was available at the MRI host computer, and therefore, these base codes were not retrievable.

As some procedures are performed very infrequently, the training data base was not large enough to learn features for the prediction of all billing codes reliably—thus, codes that occur in less than 20 instances of the training data (i.e. in less than 0.3%) were discarded. The distribution of removed billing codes within the dataset is presented in the “Results” section of this paper.

As found out during the analysis of the billing codes, coding errors were present in the available data. Errors that violate Tarmed coding rules were corrected automatically in the training dataset of the algorithm: if codes were charged more than the maximum allowed number per exam, the number of the code was reduced to the allowed quantity. This enhances the quality of the target data, although no ground truth level was reached as coding errors remain in the data that can only be corrected through manual analysis of the billing codes. The manual correction of the codes was limited to the test dataset (see the “Generation of ground truth data” section).

### Prediction model: automated procedure coding

#### Feature extraction

Features were extracted from the set of executed sequences during the MRI exam, associated sequence parameters (e.g. field-of-view (FOV)), table movements of the MRI scanner table, used coils, and approximate age of the subject. Moreover, information whether the contrast medium (CM) was used was incorporated into the feature set.

The total acquisition time, ACQ, (i.e. sum of sequence execution time or value-added time according to Lean Six Sigma [14, 15]) and the user-operating time, UOP, (idle time of the scanner, non-value-added time according to Lean Six Sigma, i.e. exam duration minus acquisition time) were computed, and the ratio of UOP-to-ACQ was calculated. The UOP-to-ACQ ratio can be used for the identification of MR-guided biopsies since acquisition cycles during MR-guided biopsies makeup only a small share of the total exam duration. During diagnostic MRI exams, the UOP-to-ACQ ratio is significantly lower.

In order to further distinguish MRI procedures, the MR scanner table movement was investigated. The MR scanner table usually does not move much or in a recognizable pattern during the exam. However, for some procedures (e.g. whole-body exam or MR-guided biopsy), the table movement has a distinct shape, and features from the table movement during the exam were extracted to identify these procedures (e.g. total table movement, min/max table position, average table movement between two sequences).

#### Sequence name standardization

The set of executed sequences provides useful information for the identification of the conducted exam, since different sequences are used for the assessment of different clinical indications. However, the names of the sequences can be adapted individually, and therefore they are not comparable and meaningful features for the characterization of an MRI exam. For instance, the (user-defined) sequence names “t1_tse_tra_fs” and “t1_tse_fs_tra” (**t1**-weighted **t**urbo **s**pin **e**cho sequence in **tra**nsverse imaging direction with **f**at-**s**aturation) falsely encode two different MRI sequences and consequently also features. Standardization will solve this problem by generating a structured sequence name.

Since sequence information is often not stored reliably in the sequence name, it complicates the use of machine learning to learn sequence name terms. Therefore, sequence terms were generated heuristically based on the underlying sequence parameters (e.g. TR, TE, TI, imaging technique, imaging orientation).

The sequence name standardization makes sequences names comparable by unifying the structure of the sequence name and by removing sequence terms that do not describe intrinsic or crucial properties of the sequence. Examples are shown in Table 1. Sequence terms in the original sequence name, which are not part of a generic, standardized sequence name, are, e.g. the specified body region (“ep2d_diff_3b_**Abdomen**”), the slice thickness (“t2_haste_fs_tra_**3mm**_mbh”), or the time after CM injection (“t1_vibe_fs_tra_caipi_**15 min**”).

**Table 1:** Examples of original and standardized sequence name pairs

Original sequence name	Standardized sequence name
ep2d_diff_3b_Abdomen	ep2d_diff tra
ep2d_diff_b50_300_800_tra_4Scan_p3	ep2d_diff tra
t2_haste_fs_tra_3mm_mbh	t2 haste fs tra bh
t1_vibe_fs_tra_caipi_15 min	t1 vibe fs tra bh

Abbreviations: ep2d = two-dimensional (2d) echo-planar imaging; diff, diffusion; tra, transverse; mbh, multiple breath hold

The first two sequences listed in the table represent the same diffusion weighted sequence and are therefore mapped to the same standardized sequence name

See http://www.revisemri.com/questions/misc/mri_abbrev for an extensive list of common MRI abbreviations that explain the remaining sequence terms

The standardization enables the utilization of sequence names as features, even across different scanners and sites, and furthermore reduces the amount of different sequence names, increasing the generalization ability of the classification model while decreasing the training time.

#### Classification

Different base classifiers were trained using the extracted features from the MRI log data and evaluated. As a base classifier, a fully connected feed-forward neural network (multilayer perceptron (MLP)), a SVM, and a random forest as base classifier were applied and compared, as they have been proven to be the best-performing classifiers for a variety of classification problems [16].

In traditional supervised learning, each instance, characterized by its features, is associated with a single label (two-class/binary learning problem). If the label can have more than two different values, it becomes a multi-class problem. In multi-label learning, each instance is associated with a set of *q* different binary labels. Furthermore, multi-output classification is a generalization of multi-label classification, where each label can be multi-class (i.e. more than two different values can be assigned to a label) [17].

The prediction of billing codes is a multi-output classification problem, since some billing codes can be multi-class, i.e. identical codes can be assigned multiple times to an MRI exam—for instance, codes corresponding to a body region of the extremities can be charged twice, or a surcharge code for additional series can be charged for each reported main service code.

It is assumed that interdependencies between billing codes exist, e.g. a biopsy procedure billing code occurs more likely in combination with an abdomen code than with a whole-body code. To cope with label dependencies, the problem transformation method ensemble of classifier chains (ECC) [18] was applied and compared to the binary relevance method [19]. Classifier chains arrange classifiers into a chain, whereas each classifier incorporates the classification output of the preceding classifiers as additional features. Ensembles of classifier chains leverage the benefit of ensemble learning by combining multiple classifier chains with random label order. The final output for each label is yielded by a majority vote of the output of the single classifier chains. In contrast, the binary relevance method constructs a single classifier for every label, and therefore independence between the labels is assumed, which makes the method computationally efficient and highly resistant to overfitting label combinations, but neglects any relationship between labels.

Other problem transformation methods, such as hierarchical classifier [5] or (pruned) labelsets [20] have not proven to be beneficiary and therefore are not further reported and assessed in the “Results” section of this paper.

### Target data descriptors and evaluation metrics

In multi-output learning, the dataset is given by *D* = {(***x***_*i*_, ***y***_*i*_)| 1 ≤ *i* ≤ *m*}, where *m* is the number of instances in the dataset. Each multi-output instance is represented by the *n*-dimensional feature vector ***x***_*i*_***=*** [*x*_*i*, 1_,  … , *x*_*i*, *n*_] and the labelset ***y***_***i***_***=*** [*y*_*i*, 1_,  … , *y*_*i*, *q*_], with *q* being the number of different labels, and *y*_*i*, *j*_ ∈ *Υ*_*j*_ = {1,  … , *K*_*j*_}, with *K*_*j*_ ∈ *ℕ*_+_ being the finite number of values associated with the *j*-th label. The multi-output classifier *h*(∙) is then trained to predict the set of labels h(**x**) = **y** for an unseen instance **x** [17, 21].

Label cardinality, density, and diversity are computed to characterize the complexity of the multi-output target data. Label cardinality quantifies the average number of codes per MRI exam. Thus, it measures the degree of multi-labeldness of the dataset and is given by

1 $$ \boldsymbol{LCard}\left(\boldsymbol{D}\right)=\frac{\mathbf{1}}{\boldsymbol{m}}\sum \limits_{\boldsymbol{i}=\mathbf{1}}^{\boldsymbol{m}}\sum \limits_{\boldsymbol{j}=\mathbf{1}}^{\boldsymbol{p}}{\boldsymbol{y}}_{\boldsymbol{i},\boldsymbol{j}}. $$

Label density is defined by the label cardinality divided by the total number of possible codes:

2 $$ \boldsymbol{LDen}\left(\boldsymbol{D}\right)=\frac{\mathbf{1}}{\left|\boldsymbol{Y} \right|}\boldsymbol{LCard}\left(\boldsymbol{D}\right). $$

Label diversity gives the number of distinct code sets in the dataset:

3 $$ LDiv(D)=\left|\Big\{\boldsymbol{y}|\exists \boldsymbol{x}:\left(\boldsymbol{x},\boldsymbol{y}\right)\in D\Big\}\right|. $$

Recall, precision and F1-score are reported for the assessment of the performance of the prediction model.

Since the prediction of billing codes is a multi-output classification problem, different averaging methods for the evaluation measures can be computed (e.g. macro- or micro- average). Let *B*(∙) represent some specific binary classification metric (e.g. *B* ∈ {precision; recall; F1}), depending on *TP*_*j*_, *FP*_*j*_, *TN*_*j*_, *FN*_*j*_, class label *j*, classifier *h*. *TP* indicate the true positives, *FP* the false positives, *TN* the true negatives, and *FN* the false positives. The macro- and micro-average of the classification metric *B*(∙) are then defined as follows [17]:

4 $$ {B}_{macro}(h)=\frac{1}{q}B\left({TP}_j,{FP}_j,{TN}_j,{FN}_j\right) $$

5 $$ {B}_{micro}(h)=\frac{1}{q}B\left(\sum \limits_{j=1}^m{TP}_j,\sum \limits_{j=1}^m{FP}_j,\sum \limits_{j=1}^m{TN}_j,\sum \limits_{j=1}^m{FN}_j,\right). $$

The macro-average assumes equal weight for each label, whereas the micro-average incorporates the frequency of the labels into the label weighting. Since some billing codes occur in less than 1% of the available data, micro-averaged scores are more suitable to assess the overall quality of the prediction model, and therefore the micro-averaged recall, precision, and F1-score are reported.

Additionally, the subset accuracy, also known as classification or labelset accuracy, is reported. The subset accuracy is given by

6 $$ SubsetAccuracy=\frac{1}{m}\sum \limits_{i=1}^m\left\Vert {\boldsymbol{y}}_i=h\left({\boldsymbol{x}}_i\right)\right\Vert . $$

### Generation of ground truth data

Since errors in the billing codes that were reported by the technologists were found (either codes were forgotten or falsely charged), the available codes cannot be considered to be ground truth data. For the final evaluation, a held-out test set was corrected exam-wise to generate ground truth billing codes. Reported procedures in the RIS, the DICOM images, and the final report stored in the PACS were used to validate the billing codes and ambiguous cases were additionally discussed with the lead technologist. Therefore, the test set establishes ground truth and can both be used to evaluate the quality of the classification model and of the billing codes manually entered by the technologists.

Due to a large amount of data (9754 training instances with over 110,000 executed MRI sequences), it was not feasible to correct the training data manually to generate ground truth. However, although the training dataset is not ground truth code data, its data quality after data cleaning was considered to be sufficient for training an algorithm.

## Results

### Data

The dataset spans a time period of 21 months, whereas the training set comprises 9754 instances and the test set comprises 423, covering a complete month of data. In total, the target data contain 28 different Tarmed codes. After the data cleaning process, 22 different codes remained, from which six can also be charged twice and two can also be charged more than twice for a single MRI exam. The removed codes account for only 0.1% of the total generated reimbursement within the dataset and are therefore neglectable.

**Fig. 2:**
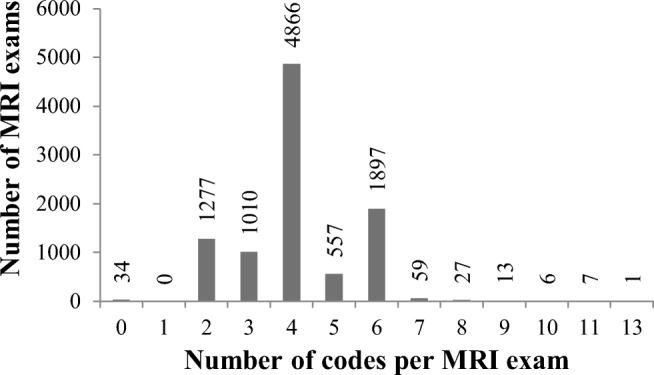
Distribution of the number of procedure codes per MRI exam in the training dataset. If no procedure code was reported for an MRI exam, it was aborted before images sufficient for diagnosing were acquired

The cardinality of the training dataset is 4.1 and the label density is 0.1. One hundred seventy-two unique combinations of these labels (labelsets) are observed in the training dataset. The label cardinality and density of the corrected test dataset are identical to the training set. Due to its smaller size, its label diversity is only 61, whereas also six labelsets in the test dataset do not occur in the training dataset. The distribution of the number of billing codes per MRI exam in the training dataset is shown in Fig. 2. A total of 98.5% of the MRI exams in the training dataset contain between two and six codes.

### Assessment of automated coding

The standardization of the MR sequence names is a crucial processing step of the prediction pipeline. Over 110,000 sequences were executed on the two MRI scanners during the MRI exams comprised by the training data, with 1002 different sequence names in total. Using the sequence name standardization, the original amount of 1002 different sequence names were reduced to 334, which is a decrease of 67%.

Binary relevance and ensemble of classifier chains were implemented with the base classifiers MLP, RBF SVM, and random forest; the hyperparameters of each model were optimized using 10-fold cross-validation on the training dataset with regard to micro F1-score.

The best-performing classifiers with regard to micro-averaged F1-score were the ECC with the multilayer perceptron (97.8%) as a base classifier and ten classifier chains. The classification results for the evaluated methods are presented in Table 2. Top recall (98.1%) was also achieved with the ECC-MLP classifier. Label cardinality and density did not differ much across the tested classifiers.

**Table 2:** Evaluation scores and label metrics for the test prediction using different classification methods

	Binary relevance			Ensemble of classifier chains	Ensemble of classifier chains
	MLP	SVM RBF	Random forest	MLP	Random forest
Micro F1-score	97.5%	95.3%	97.3%	97.8%	97.5%
Micro precision	97.4%	96.4%	97.7%	97.5%	97.5%
Micro recall	97.6%	94.3%	97.0%	98.1%	97.5%
Subset accuracy	90.8%	84.6%	90.6%	91.7%	91.3%
Label cardinality	4.1	4.0	4.1	4.1	4.1
Label density	0.2	0.2	0.2	0.2	0.2
Label diversity	57	40	47	55	55

### Assessment of manual coding

The technologists’ manual coding performance reached micro-averaged F1-score of 98.1% and a subset accuracy of 92.0%, slightly surpassing the performance of automated coding. The precision of manual coding was at 98.8% and recall at 97.4%, which is inferior to the recall of the MLP-based ensemble of classifier chain (98.1%). Label cardinality and density are equal to the ground truth dataset; label diversity is 67, thus higher than of the ground truth dataset.

**Fig. 3:**
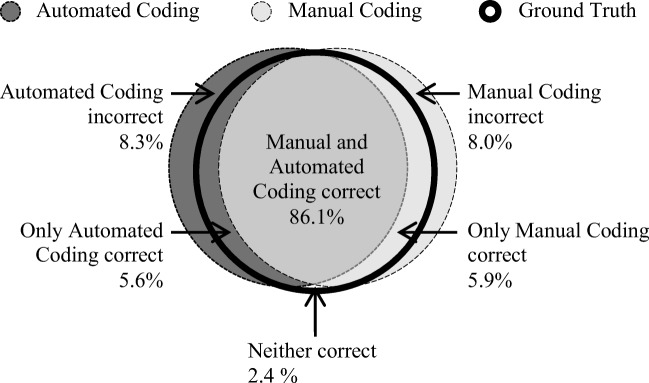
The Venn diagram illustrates the performance difference of automated and manual coding with regard to the subset accuracy. Here, the ECC-MLP model was used for automated coding. In a significant share of the test instances, automated coding was superior to manual coding (5.6%)

Although micro-averaged F1-score and subset accuracy of manual coding were still superior to automated coding, in a significant share of test instances (5.6%) automated coding was correct, whereas manual coding was incorrect (see Fig. 3).

## Discussion

The results above show not only that manual coding is prone to errors but also that automated coding has a performance that is similar to the performance of manual coding. In the following, coding performance is discussed in more detail, and an outlook for automated coding for MRI exams is given.

### Automated coding

Performance comparison to other automated coding methods is difficult, since most methods as outlined in the “Related work” section focus on diagnosis coding. Moreover, the performance of published methods must always be considered in the context of the underlying complexity in terms of the used data basis and coding system [22]. One of the few works on automated procedure coding presented in [11] yielded a F1-score of 48.5% on the prediction of ICD-10-PCS codes from narrative text. This is inferior to the performance of this work, which shows the value of using modality log data for the retrieval of billing codes over narrative text as data basis. However, since no subset analysis for imaging or MRI billing codes is provided and different coding systems are applied, a thorough performance comparison cannot be made.

In order to evaluate the possibilities for future performance improvements, we assessed the relationship between code prevalence in the training data and F1-score. Therefore, we computed the Spearman rank correlation coefficient that is a nonparametric measure to quantify the statistical dependency between two variables. We computed the coefficient between the F1-score and code prevalence for all codes that yield imperfect results (i.e. F1-score below 100% on the test dataset). The coefficient is 0.89 with a *p* value of < 0.0001, showing a very strong monotonic relationship between the F1-score and the code prevalence in the training data. This indicates that the F1-score can potentially be further increased by incorporating more training data.

Furthermore, the impact of errors in the test set on the final performance evaluation was assessed by computing the performance metrics for the prediction of the ECC-MLP classifier with respect to the uncorrected, manual coding test dataset. In this case, the F1-score was underestimated by 1.3% and the subset accuracy even by 4.7%, which shows the importance of having ground truth data available for the final evaluation of the prediction model.

### Manual coding

The precision of manual coding (98.8%) was distinctively higher than its recall (97.4%), which can be explained by the fact that codes were usually rather forgotten by the technologists to be charged than accidentally entered. Moreover, 63% of manual coding errors were due to Tarmed auxiliary codes 39.5010, 39.0410, and 39.5020 (constituting 45% of the total number of assigned codes), which were added manually to the main procedure service (i.e. the billing code for an additional MRI series, arthrogram, or angiography). This manual process step is the cause of the increased error rate. Thus, coding aide focusing on auxiliary codes could help reduce coding errors considerably. Moreover, coding errors within the test set occurred most often for neck exams, which is likely due to the high number of billing codes per exam (average number of six codes) that increases the complexity of the coding process. The remaining errors occurred evenly distributed across all types of MRI exams. This indicates that in general manual coding errors occur due to codes being added or forgotten through an oversight, rather than due to insufficient coding knowledge of the technologists for specific types of MRI exams.

However, it is anticipated that coding performance varies across technologists, depending, e.g. on their work experience. Due to personal data protection issues, these data were not collected and consequently, the variance of the performance between single technologists could not be measured.

We also assessed the impact of coding errors on generated reimbursement. With respect to the ground truth billing code data, coding errors by the technologists would have resulted in a loss of 2.9% of reimbursement due to manual undercoding errors. On the other hand, overcoding errors by the technologists would translate into falsely charged, an additional 0.8% of reimbursement. Due to increasing cost pressure in healthcare [21, 22], it is crucial to charge all rendered procedures and services correctly by avoiding any procedure coding errors. This can either be achieved with the current process through a labor-intensive additional manual review or through coding support by using automated procedure coding.

### Potential application

The model was developed with the primary focus to enhance the billing process for MRI exams. Medical billing describes the process of submitting claims to the insurer that comprise reported billing codes and consumed materials. Different applications of this work are conceivable in the radiology environment that can enhance the workflow. For instance, the prediction model can be employed as a standalone tool to support the technologist during medical procedure coding in form of suggesting likely billing codes for an MRI exam to ensure that all rendered services are reported.

If the prediction model is supposed to be used in a semi-automated (billing) setting, DICOM services (such as DICOM Modality Performed Procedure Step (MPPS) [23]) could be utilized as an interface between the MRI host computer/scanner and the RIS that allows the transfer of billing codes. Since medical billing does not only comprise encoding procedures but also consumed materials, this type of billing/coding support is only semi-automated. Materials used for MRI exams, e.g. syringes for the injection of contrast medium or needles for MR-guided biopsies, are also reported in the RIS for billing. However, the automated reporting of used materials has not been investigated within the scope of this paper. Additionally, current non-imaging related codes prevent a fully automated procedure coding and consequently also automated billing.

### Limitations and future works

In order to explore the possibilities for (fully) automated procedure coding and enhanced billing, material usage, better information retrieval for current unpredictable codes and algorithmic improvements shall be part of further research.

We used MRI logs as a data basis for our algorithm, which are proprietary and vendor specific. A standardized, non-proprietary alternative data basis for automated procedure coding for MRI exams could be DICOM metadata, comprising information that offers comparable content. However, challenges with regard to processing DICOM metadata may include efficient data retrieval from the PACS [23] and the completeness of technical information from DICOM metadata for feature processing. Therefore, future work shall investigate how this work is transferable to DICOM metadata.

Until now, the prediction model has been tested on data from two MRI scanners of a single site. The applicability of the prediction model to data of more than one site shall be evaluated in future work. Additionally, the performance of the prediction model on different medical procedure billing systems shall be investigated. It is reasonable to expect that the model is applicable to other procedure coding systems without major adaption as most systems have a similar coding structure (e.g. modality-based, body region, and procedure-specific coding).

Moreover, the presented work may also be transferred to other medical (imaging) modalities by utilizing modality event logs to predict billing codes for billing purposes. For instance, the examined anatomy of interventional X-ray exams was already successfully retrieved from modality log data in [13]. Extending this work by training a prediction model with corresponding billing code data and tailoring the feature extraction process to this imaging modality may enable automated procedure billing coding for interventional X-ray systems. Additionally, automated procedure coding from modality log data may also be transferable to other imaging modalities, such as computed tomography.

## Conclusion

Medical procedure coding is the basis for reimbursement and therefore crucial for the financial situation of the clinical site. At the investigated radiology site, procedure coding for MRI exams is still based on manual input from the technologist and thus prone to user errors. The billing codes are currently reviewed manually by the responsible person for procedure coding to prevent coding errors, which is a time- and cost-intensive process. In this paper, we presented a method for automated procedure coding for MRI exams on the basis of MRI log data. We developed a method for sequence name standardization that increases both the generalization ability and training speed of the prediction model. We showed that automated coding, requiring no user input, reached almost the same performance (micro F1-score, 97.8%) as manual coding through the technologist (micro F1-score, 98.1%).

Thus, automated procedure coding has the potential to optimize reimbursement and reduce the workload for the technologists. It is therefore anticipated to support digitalization and workflow optimization in the radiology department.
